# Case Report: Continuous block-and-replace strategy with osilodrostat in a patient with cyclic Cushing’s syndrome

**DOI:** 10.3389/fendo.2026.1788064

**Published:** 2026-07-09

**Authors:** Wiktoria Suchy, Mari Minasyan, Aleksandra Gamrat-Żmuda, Alicja Hubalewska-Dydejczyk, Aleksandra Gilis-Januszewska

**Affiliations:** Chair and Department of Endocrinology, Jagiellonian University Medical College, Krakow, Poland

**Keywords:** bilateral inferior petrosal sinus sampling, block-and-replace therapy, case report, cyclic Cushing’s syndrome, osilodrostat

## Abstract

Evidence on cyclic Cushing’s syndrome (cCS) is limited. Due to its rarity and diagnostic challenges, some patients remain undiagnosed and experience life-threatening episodes of hyper- and hypocortisolemia. We present a case of a 68-year-old male with a 5-year history of recurrent hospitalizations due to infections, sepsis, and episodes of blood pressure and glycemic instabilities, who was admitted to the Endocrinology Department for evaluation of bilateral adrenal incidentalomas. Physical examination revealed cushingoid features, and biochemical tests confirmed ACTH-dependent CS, with dynamic tests suggesting ectopic origin. Retrospective review showed several peaks of hypercortisolemia separated by spontaneous remissions. These fluctuations correlated with severe infections, hypertension, and hyperglycemia, followed by hypotension and improved metabolic control. Medical therapy with steroidogenesis inhibitors in a block-and-replace regimen resulted in sustained biochemical and clinical stabilization. Imaging failed to identify the ACTH source; therefore inferior petrosal sinus sampling is planned to definitively establish the etiology of CS. This case illustrates the unpredictable nature of cCS and the considerable challenges it poses for both diagnosis and management, emphasizing the need for individualized therapeutic strategies.

## 1 Introduction

Evidence on the cyclic nature of Cushing’s syndrome (CS) remains limited, leading to diagnostic delays and potentially life-threatening fluctuations in cortisol secretion. Recent studies suggest defining cyclic CS (cCS) as an endogenous CS with the presence of cyclicity confirmed by at least two peaks of biochemical hypercortisolism separated by at least one phase of spontaneous remission, or even hypocortisolemia (trough) (1). Research shows cCS might be an underdiagnosed entity, with 14-18% of CS patients meeting the criteria of cCS (1). Due to unpredictable cyclicity, often life-threatening, continuous block-and-replace medical therapy represents a safe and effective treatment option in several cCS cases (2–4). To our knowledge, this is one of the first descriptions of the use of continuous block-and-replace therapy with osilodrostat in a patient with cCS.

## 2 Case presentation

We present a case of a 68-year-old male with a 5-year history of multiple hospitalizations due to recurrent severe infections as well as severe fluctuations in blood pressure and glycemic control. His medical history was notable for significant cardiovascular comorbidities and osteoporosis. The patient was admitted to the Endocrinology Department for hormonal assessment of bilateral adrenal incidentalomas (18 mm on the right and 10 mm on the left, both with benign radiological features). Physical examination revealed abdominal obesity, proximal muscle wasting, moon face, dorsocervical fat pad, skin bruises, fragile skin, face and chest plethora. At admission, hypertension was poorly controlled, with systolic blood pressure reaching 220 mmHg despite intensified therapy. The patient also reported poor sleep quality and demonstrated cognitive impairment on psychological testing. Laboratory evaluation showed marked metabolic disturbances, including severe hypokalemia despite supplementation (2.47 mmol/l), poor glycemic control, and dyslipidemia (**Table 1A**). Further biochemical evaluation showed absence of circadian cortisol rhythm, with no suppression of the cortisol secretion in 1 mg dexamethasone suppression test (DST), confirming hypercortisolism. Plasma adrenocorticotropic hormone (ACTH) concentrations were not suppressed, suggesting ACTH-dependent CS. Dynamic tests pointed to its ectopic nature (**Table 1B**).

**Table 1:** Laboratory results at the time of first admission to the Endocrinology Department.

Test	Result	
A. Basic laboratory tests		
Total cholesterol	6.9 mmol/l (N: 3.2-5.2)	
LDL cholesterol	4.2 mmol/l (N: <3.4)	
Potassium	2.47 mmol/l (N: 3.5-5.1)	
HbA1c	11.8% (N: <5.7)	
B. Hormonal tests		
Serum cortisol concentration (6:00)	21.6 µg/dl (N: 4.82-19.5)	
Serum cortisol concentration (8:00)	24.8 µg/dl (N: 4.82-19.5)	
Serum cortisol concentration (24:00)	18.9 µg/dl (N: <7.5)	
Serum ACTH concentration	75.5 pg/ml (N: 6.0-56.0)	
Serum cortisol concentration after 1 mg DST	22.5 µg/dl (N: <1.8)	
Serum cortisol concentration after 8 mg DST	20.8 µg/dl (18% decrease)	
Serum cortisol concentration in desmopressin stimulation test	-15 min	23.6 µg/dl
	0 min	22.6 µg/dl
	15 min	20.9 µg/dl
	30 min	19.4 µg/dl
	45 min	18.1 µg/dl
	60 min	17.8 µg/dl
Serum ACTH concentration in desmopressin stimulation test	-15 min	36.4 pg/ml
	0 min	33.8 pg/ml
	15 min	30.1 pg/ml
	30 min	31.1 pg/ml
	45 min	31.0 pg/ml
	60 min	29.4 pg/ml

ACTH, adrenocorticotropic hormone; DST, dexamethasone suppression test; HbA1c, glycated hemoglobin; LDL, low-density lipoprotein.

Retrospective analysis of the patient’s medical records over the previous five years revealed seven severe clinical episodes of hypercortisolemia separated by spontaneous remissions. Cortisol peaks with cortisol concentration 34.43-43.5 ug/dl were associated with severe hypertension, hyperglycemia, and infectious complications requiring hospitalization, whereas trough phases (cortisol concentration 8–13 ug/dl) coincided with clinical and metabolic improvement necessitating de-escalation of therapy. Twice, when the diagnosis of CS had been presumed, the patient had been evaluated by an endocrinologist, however, biochemical evaluation did not confirm hypercortisolism, likely reflecting testing during trough phases.

**Figure 1:**
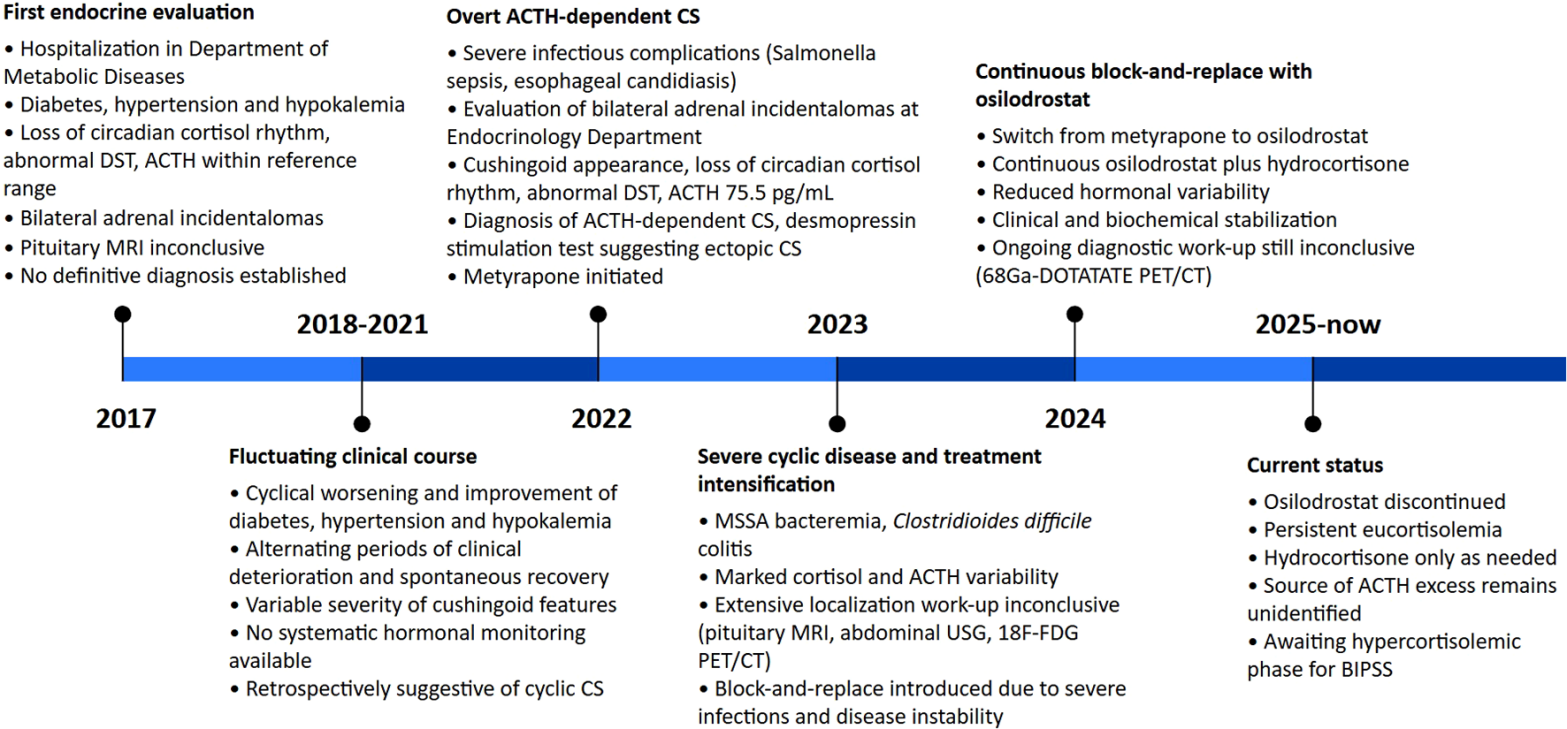
Simplified clinical timeline of the patient’s disease course. ACTH, adrenocorticotropic hormone; BIPSS, bilateral inferior petrosal sinus sampling; CS, Cushing’s syndrome, DST, dexamethasone suppression test; MRI, magnetic resonance imaging; MSSA, methicillin-sensitive Staphylococcus aureus; PET/CT, positron emission tomography/computed tomography.

**Figure 2:**
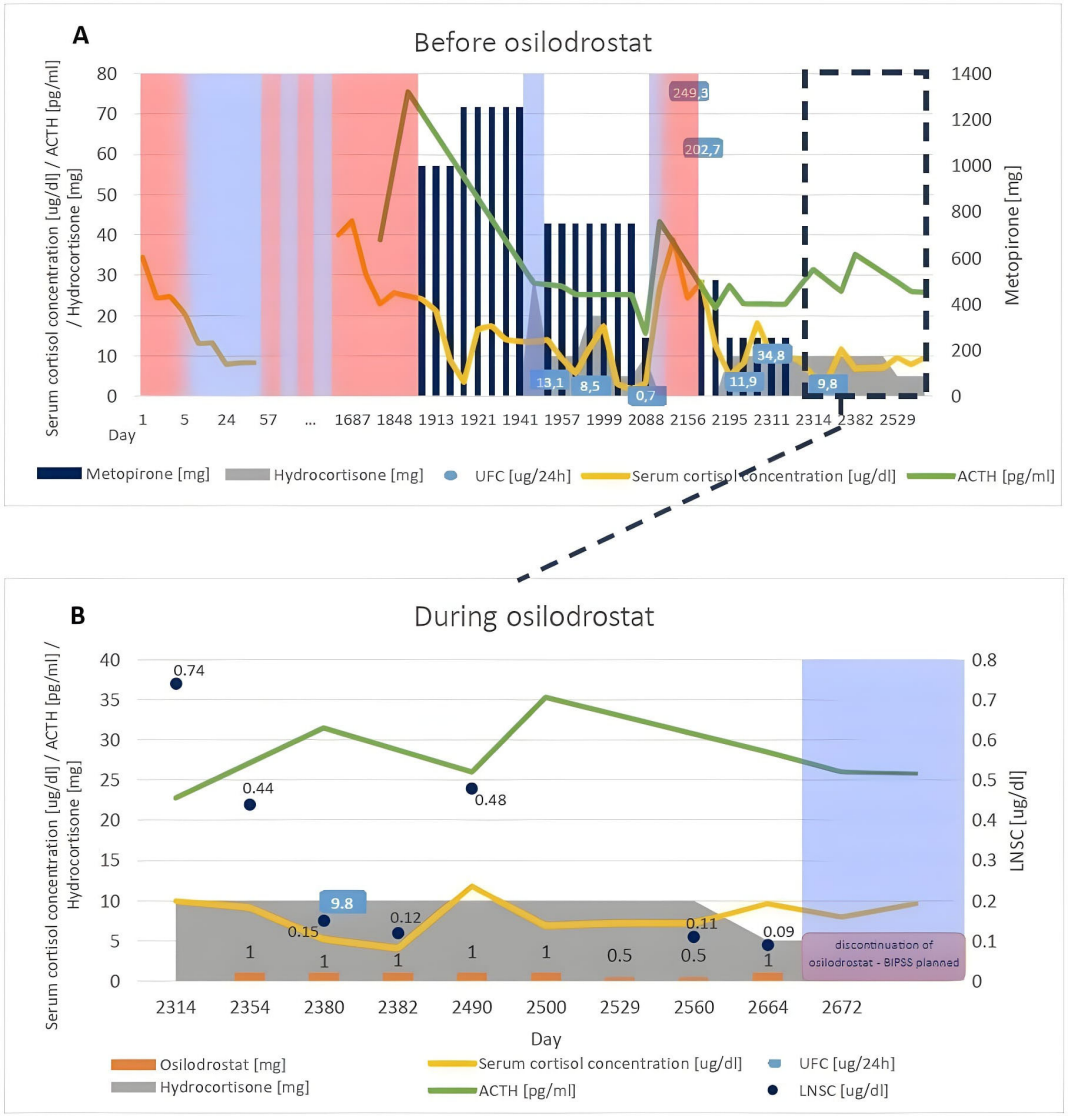
Chronological overview of hormonal fluctuations and treatment interventions before **(A)** and during osilodrostat therapy **(B)**. Shaded areas indicate presumed hypercortisolemic (red) and normo-/hypocortisolemic (blue) phases based on available clinical and biochemical data. ACTH, adrenocorticotropic hormone; BIPSS, bilateral inferior petrosal sinus sampling; LNSC, late night salivary cortisol; UFC, urinary free cortisol.

Additionally, the patient had previously been referred to an immunology clinic due to recurrent infections, an increased tendency to develop skin petechiae, and lymphopenia. After analysing the patient’s past medical history, with biochemical confirmation of two peaks (defined by 1 mg DST and clinical presentation) and one spontaneous trough (defined by morning serum cortisol and clinical presentation), diagnosis of cCS was made, with a phase of hypercortisolemia at the time of first admission to the Endocrinology Department. To achieve biochemical control, a steroidogenesis inhibitor - metyrapone, was implemented. **Figures 1**, **2** illustrate the clinical course and treatment modalities.

Hydrocortisone substitution was added to the adrenostatic therapy (block-and-replace therapy) temporarily, twice at the time of biochemical and clinical hypocortisolemia. The patient needed several dosage adjustments due to insufficient control of cortisol fluctuations, varying from hypo- to hypercortisolemic episodes, with metyrapone administered at 250–1500 mg daily and hydrocortisone at 5–30 mg daily. His morning serum cortisol concentrations showed extreme fluctuations, ranging from values typical of adrenal insufficiency (1.93 µg/dl) to those characteristic of CS (39 µg/dl), with similar variability observed in late-night salivary cortisol (LNSC) (0.11–2.15 µg/dl, N: <0.274 µg/dl) and 24-hour urinary free cortisol (UFC) (0.7–249 µg/24 h, N: 10-100 µg/24h). In total, the patient was on metyrapone for 13 months, temporarily in a block-and-replace scheme. Given the extreme and unpredictable cortisol fluctuations, causing difficulties in determining the dose of metyrapone, and due to current change in steroidogenesis inhibitors availability, during one of the troughs, the drug was switched to osilodrostat at a dose of 1 mg in the evening in a constant block-and-replace strategy (with hydrocortisone 5–10 mg daily). The dose, lower than the typical starting regimen of osilodrostat (2 mg twice daily), was based on the most recent cortisol measurements (9.96 and 10.4 µg/dl), allowing for a less escalated treatment approach. The switch was also motivated by the more favorable pharmacokinetic profile of osilodrostat. In contrast to metyrapone therapy, during which hydrocortisone supplementation was administered only intermittently in periods of increased glucocorticoid demand (e.g., infections), hydrocortisone was introduced as a continuous safety strategy for anticipated trough phases.

Monitoring of the treatment was based on morning serum cortisol concentration measured prior to hydrocortisone intake, since the UFC may be unreliable in the block-and-replace setting due to interference with exogenous glucocorticoids (5). Decisions regarding dose adjustments were made based on these measurements in conjunction with the patient’s clinical presentation and symptoms suggestive of either hyper- or hypocortisolism. As for the safety monitoring, the patient underwent regular follow-up visits with blood pressure assessment, laboratory evaluation for electrolyte disturbances, and monitoring for symptoms suggestive of adrenal insufficiency. This regimen resulted in sustained biochemical control and marked clinical stabilization over one year of follow-up, including improvement in overall well-being, sleep quality, cognitive function, and quality of life. No adverse effects related to the treatment were observed, and the patient did not experience any serious infections or severe fluctuations of hyperglycemia or hypertension which required hospitalization. He currently exhibits improved metabolic control and stable body weight. Blood pressure control improved, with values at home ranging between 115-130/60–80 mmHg. Glycemic control also improved, with fasting home glucose measurements ranging from 90–115 mg/dl. Changes in cardiometabolic parameters and concomitant treatment are summarized in **Table 2**.

**Table 2:** Cardiometabolic and therapeutic changes during osilodrostat treatment.

Variable	Before	After
A. Basic laboratory tests		
Total cholesterol	6.9 mmol/l (N: 3.2-5.2)	2.6 mmol/l (N: 3.2-5.2)
LDL cholesterol	4.2 mmol/l (N: <3.4)	0.64 mmol/l (N: <3.4)
Potassium	2.47 mmol/l (N: 3.5-5.1)	5.1 mmol/l (N: 3.5-5.1)
HbA1c	11.8% (N: <5.7)	5.6% (N: <5.7)
B. Therapeutic changes		
Antihypertensive drugs	Amlodipine 10 mg Ramipril 10 mg Metoprolol 100 mg Torasemide 10 mg Doxazosin 4 mg Nitrendypine 10 mg	Lercanidipine 20 mg Ramipril 5 mg Metoprolol 50 mg Torasemide 10 mg Doxazosin 2 mg
Antidiabetic drugs	Metformin 2000 mg Empagliflozin 10 mg Multiple daily injections of rapid-acting insulin analog Once-daily injection of a long-acting insulin analog	Metformin 1000 mg Empagliflozin 10 mg
Lipid-lowering drugs	-	Rosuvastatin 5 mg Ezetimibe 10 mg
Potassium supplementation	60 mEq/day	10 mEq/day

HbA1c, glycated hemoglobin; LDL, low-density lipoprotein.

Extensive multimodal imaging, including pituitary MRI and whole-body functional imaging with [68Ga]Ga-DOTATATE PET/CT and [18F]FDG PET/CT, failed to localize the source of ACTH overproduction. Abdominal ultrasound demonstrated a 12×12 mm hypoechoic lesion located at the border of the pancreatic body and tail. However, both [68Ga]Ga-DOTATATE PET/CT and [18F]FDG PET/CT showed no evidence of a metabolically active pancreatic lesion. The patient is currently awaiting control abdominal MRI to further evaluate the lesion, potential source of ACTH overproduction in this localization. Pituitary MRI showed mildly heterogeneous enhancement without a distinct focal lesion. Bilateral inferior petrosal sinus sampling (BIPSS) is therefore planned to verify the potential pituitary etiology of ACTH-dependent CS. Since this procedure requires a hypercortisolemic phase for optimal accuracy, adrenostatic therapy was temporarily discontinued, with the patient maintained on hydrocortisone replacement, currently only on an *ad hoc* basis.

## 3 Discussion

Cyclic Cushing’s syndrome poses a significant challenge to clinicians due to its unpredictability, with patients experiencing alternating, sometimes life-threatening episodes of hyper- and hypocortisolemia. To date, only limited data have been available. However, this topic is now gaining increasing recognition, particularly with the first international and largest observational study on cCS by Nowak E. et al., in which our center participated (2).

As in noncyclic CS, Cushing’s disease is the most common cause of cCS, accounting for 67% of all cases, however it also occurs in ectopic CS (26%) and adrenal tumors (11%) (1). In our case, dynamic tests suggested an ectopic source of ACTH excess, however there is still no definitive diagnosis. The patient demonstrated irregular, several-month-long cycles, while in published cases they ranged from a few hours (6) to many months (7), or even years (8), with irregular phase lengths being more commonly reported than regular ones (1). Previous studies have shown that ectopic and occult cCS are associated with the most pronounced cortisol fluctuations and a higher prevalence of hypokalemia (2).

Our patient with an occult presentation demonstrated similarly marked and clinically relevant cortisol variability. Cyclicity is likely underrecognized in clinical practice, since fluctuating cortisol concentrations may lead to normal results during trough phases, causing diagnostic uncertainty and delayed recognition. As evidenced in the recent retrospective study, 41% of patients with cCS had a delayed diagnosis and 43% had a delayed introduction of treatment (2), which was also the case in our patient, whose diagnosis and treatment were established after five years. This, combined with therapy-induced biochemical remission achieved in only 50% of cCS patients after 5.8 years of follow-up, highlights the considerable challenges posed by this condition (2).

Mechanisms underlying cortisol cyclicity remain incompletely understood. Proposed explanations include hypothalamic dysfunction leading to intermittent ACTH release (9–11), alterations in hypothalamic-pituitary-adrenal axis regulation (12–14), and, in some cases, structural changes within pituitary adenomas such as intermittent necrosis or hemorrhage (15, 16). These mechanisms may coexist and likely contribute to the marked heterogeneity of cyclic patterns and clinical presentation observed in cCS. Diagnosis of cCS requires high clinical awareness and repeated biochemical assessment, as standard single-timepoint tests may be falsely negative during remission phases. UFC and LNSC are particularly useful, whereas DST may show impaired diagnostic accuracy (6, 17). Hair cortisol analysis may further complement standard testing by providing a retrospective assessment of cortisol exposure (18, 19).

BIPSS has been widely recognized as an excellent tool in the differential diagnosis of ACTH-dependent CS (1). However, it needs to be done in the hypercortisolemic phase, as performing it during the trough phase increases the rate of false negative results, extending the time to diagnosis and exposing the patient to avoidable risk (5, 20, 21). Accurate timing of BIPSS in cCS is therefore essential to achieve reliable results and guide further management. This issue is particularly challenging in patients with long-term remissions, which was also the case in our patient, who has remained in a trough phase throughout an 11-month follow-up after steroidogenesis inhibitor discontinuation, making it impossible to perform a reliable BIPSS.

In our patient, the fluctuating hypercortisolemia had a profound effect on his comorbidities. The patient had severe hypertension, hypokalemia, glycemic instability, dyslipidemia, osteoporosis, and recurrent infections. Notably, the cyclical pattern of hypercortisolism was reflected clinically by alternating periods of metabolic decompensation and stabilization, with hospitalizations often coinciding with presumed cortisol peaks, even without biochemical confirmation. This emphasizes the contribution of cortisol cycling to marked cardiovascular and metabolic instability, including blood pressure variability, arrhythmic risk in the setting of hypokalemia, and increased hospitalization burden. Consistent with noncyclic CS, surgery is the preferred treatment of cCS, depending on the etiology. However, when surgery is contraindicated or in patients with occult tumor, as in our patient, pharmacotherapy remains a viable strategy. In patients with severe and unpredictable cortisol fluctuations, continuous block-and-replace therapy represents a pragmatic and safer alternative to dose titration. This regimen’s rationale is to induce hypocortisolism through steroidogenesis inhibitor treatment, followed by glucocorticoid replacement, which allows for better predictability of cortisol concentrations, and is particularly useful when close monitoring is not achievable (1, 3–5). Worth mentioning is the fact that there is an ongoing randomized clinical trial evaluating block-and-replace therapy with osilodrostat and concomitant glucocorticoid replacement in patients with endogenous hypercortisolism (NCT06430528). This study could provide further insights into the long-term management of this regimen.

In our patient, the severe and cyclic nature of hypercortisolism, combined with the presence of an occult tumor, made the block-and-replace strategy particularly appropriate, allowing for more stable cortisol control in a clinically challenging setting. While the role of block-and-replace therapy in severe hypercortisolism has been widely reported, less is known about the utility of this approach in cCS. Although this regimen has been proposed as a useful approach in cCS, our case represents one of the first descriptions of continuous block-and-replace therapy with osilodrostat in a patient with cCS, providing real-world insight into the practical challenges of managing marked cortisol fluctuations.

One of the limitations of this study is the still unknown source of ACTH overproduction. The patient’s current eucortisolemic phase precludes performing a reliable BIPSS. This reflects a well-recognized challenge in ACTH-dependent CS, where the underlying source may remain occult despite extensive diagnostic work-up. Importantly, this case highlights the need to balance ongoing diagnostic investigations with timely therapeutic intervention. In patients with severe cyclic hypercortisolism, diagnostic uncertainty should not delay treatment, as recurrent cortisol excess may lead to significant cardiovascular, metabolic, and infectious morbidity. In this context, medical therapy may act as a stabilizing strategy while awaiting definitive etiologic localization. Furthermore, this case highlights the diagnostic complexity of cCS and the importance of increased clinical awareness to reduce diagnostic delays and associated complications. Continuous block-and-replace therapy with osilodrostat may provide sustained biochemical control and clinical stability in patients with highly unpredictable cortisol secretion, particularly when the ACTH source remains occult. Our observations support the potential utility of this therapeutic regimen in carefully selected patients, where conventional management strategies may be insufficient.

## Data Availability

The original contributions presented in the study are included in the article/**Supplementary Material**. Further inquiries can be directed to the corresponding author/s.
