# Supplementary figures and images for: Case Report: Continuous block-and-replace strategy with osilodrostat in a patient with cyclic Cushing’s syndrome
Source: Front Endocrinol (Lausanne). 2026 Jul 9;17:1788064. doi: 10.3389/fendo.2026.1788064 (PMC13391415; doi:10.3389/fendo.2026.1788064)

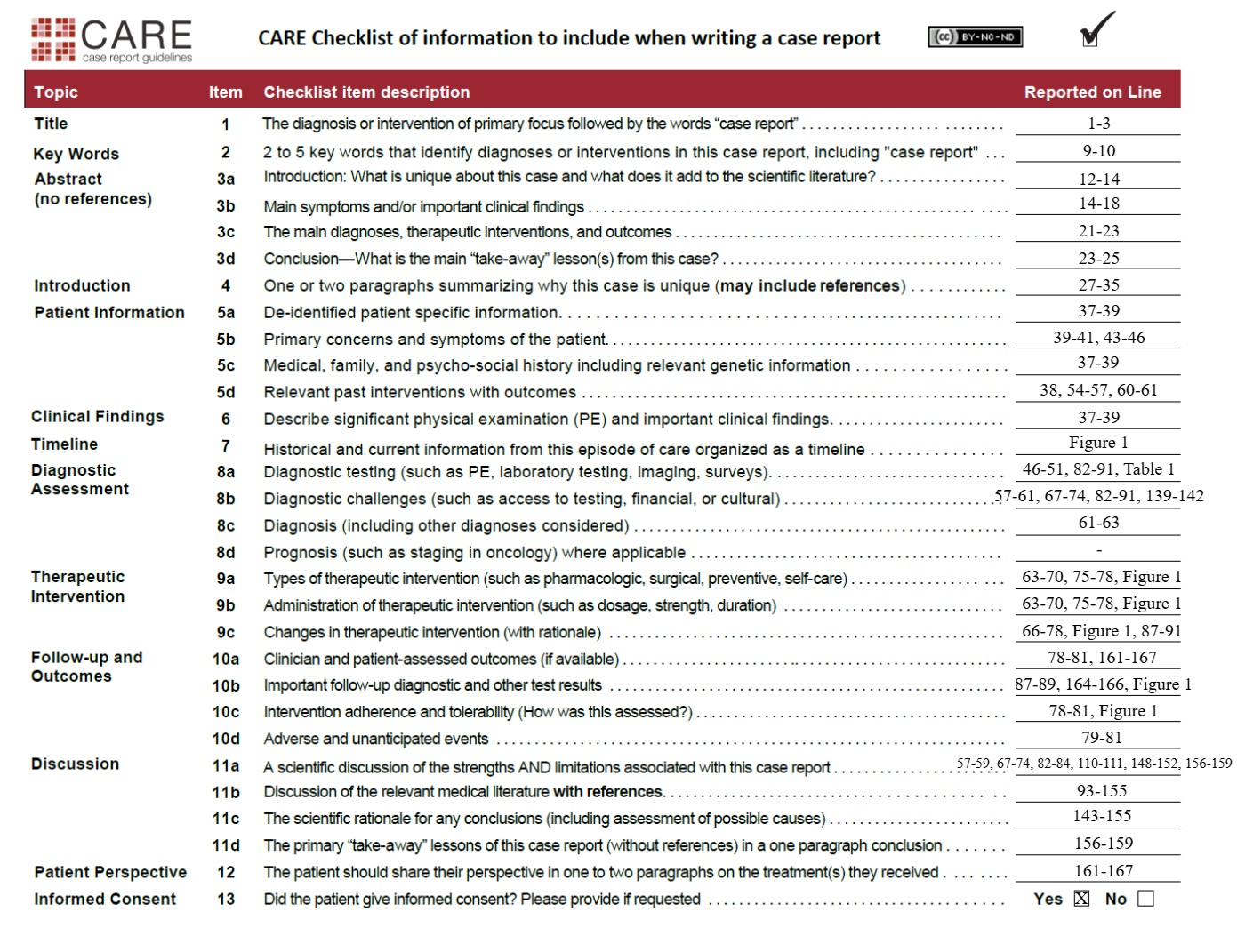

Supplement: Supplementary file 1 [file Image1.jpeg]
